# Atypical Ocular Toxoplasmosis: Multifocal Segmental Retinal Arteritis (Kyrieleis Arteritis) and Peripheral Choroidal Leision

**DOI:** 10.7759/cureus.47060

**Published:** 2023-10-15

**Authors:** Joobin Khadamy

**Affiliations:** 1 Ophthalmology, University Hospital of Umeå, Umeå, SWE

**Keywords:** segmental retinal arteritis, multifocal segmental arteritis (kyrieleis arteritis), kyrieleis arteritis, infectious uveitis, iris atrophy, frosted branch angiitis, choroidal granuloma, ophthalmologic findings of ocular toxoplasmosis

## Abstract

A 45-year-old immunocompetent man with a two-week history of unilateral painful red eye was referred to the university hospital for further investigation. High intraocular pressure, corneal edema, large pigmented keratic precipitates, cells and flares in the anterior chamber, patches of iris transillumination with atrophy, multifocal segmental retinal arteritis (SRA) or Kyrieleis arteritis, and peripheral choroidal elevation with overlying vitritis without adjacent old scars were observed. Toxoplasmosis, varicella-zoster virus, herpes simplex, and cytomegalovirus serologies (IgG) were positive. More detailed history-taking revealed that the patient consumed grilled rats in Ghana where rats are eaten more regularly. Toxoplasmosis diagnosis was assumed. The patient was successfully treated according to local guidelines with azithromycin 500 mg/day for five weeks. No recurrence or new lesion was observed during the six-month follow-up period.

## Introduction

Ocular toxoplasmosis, which results from infection with the obligate intracellular protozoan parasite Toxoplasma gondii, ranks as one of the leading contributors to chorioretinal inflammation, visual impairment, and a heightened likelihood of recurring episodes. Frequently, there is the presence of mild to moderate granulomatous anterior uveitis, and as many as 20% of patients may experience an acute increase in intraocular pressure (IOP) when initially diagnosed. The diagnosis of ocular toxoplasmosis is classically based on the finding of characteristic clinical findings, which include focal chorioretinitis, an adjacent chorioretinal scar, and moderate to severe vitritis. While these lesions are more frequently observed in the posterior pole of the eye, they can occasionally be found in close proximity to or even directly affecting the optic nerve, potentially leading to confusion with optic neuritis. However, "atypical" presentations of the disease may be challenging for clinicians, delaying proper diagnosis and timely treatment. Immunocompromised or elderly patients may, for instance, present with large, multiple, and bilateral lesions. Other unusual manifestations include punctate outer retinal toxoplasmosis, retinal vasculitis, vascular occlusions, retinal detachments, frosted branch angiitis, Kyrieleis arteritis, Coats’-type response, Fuchs’-like anterior uveitis, multifocal diffuse necrotizing retinitis, unilateral pigmentary retinopathy mimicking retinitis pigmentosa, neuroretinitis and papillitis, and scleritis [1].

In the current report, we depict an atypical primary ocular toxoplasmosis presentation with multifocal Kyrieleis arteritis and peripheral choroidal lesion. We discussed the challenges that we encountered during the management of the patient. 

## Case presentation

A 45-year-old immunocompetent man presented with a two-week history of unilateral painful red eye, returning from Ghana. Initial examination revealed hand motion (HM) vision, high intraocular pressure (IOP= 63 mmHg), corneal edema, and hazy fundus. The patient was referred from another center to us due to suspicion of herpes uveokeratitis and was on oral valacyclovir 500 mg three times a day. This was discontinued after primary evaluation.

**Figure 1:**
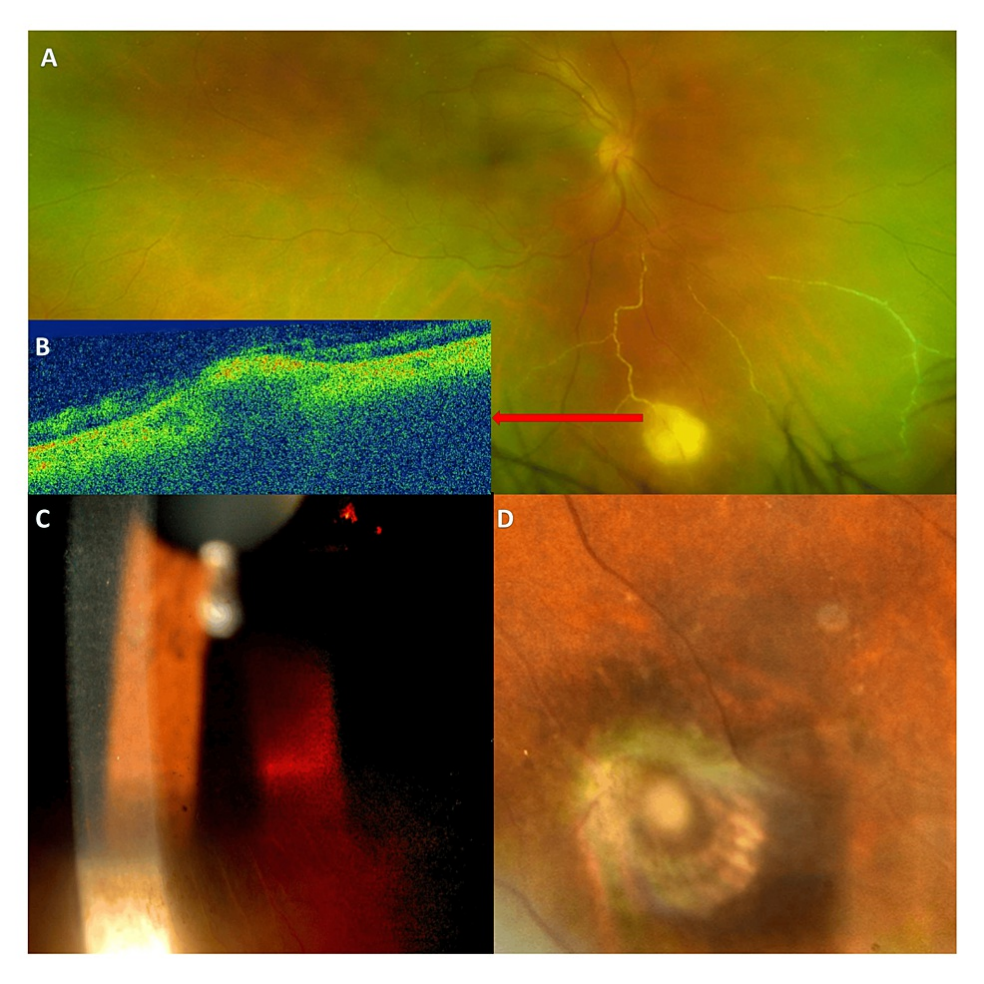
Atypical ocular toxoplasmosis: multifocal Kyrieleis arteritis, peripheral chorioretinal lesion, and iris atrophy A: Widefield fundus image shows multifocal Kyrieleis arteritis with segmental intravascular yellow-white Kyrieleis plaques and nearby single peripheral chorioretinal lesion with overlying vitritis without adjacent old scars. B: OCT B-scan section from the chorioretinal lesion shows a hyperreflective choroidal elevation with posterior hyporeflectivity and thinning of the overlying retina resembling choroidal granuloma. C: Slit lamp photo of the anterior segment shows large pigmented keratic precipitates and patches of iris transillumination with atrophy. D: Chorioretinal atrophy and regression of arteritis five weeks after treatment with azithromycin monotherapy.

After IOP reduction applying topical eye drops, a more detailed inspection showed large pigmented keratic precipitates (KP), cells (3+) and flares (3+) in the anterior chamber (A/C), patches of iris transillumination with atrophy, multifocal Kyrieleis arteritis with segmental intravascular yellow-white Kyrieleis plaques, and single white peripheral chorioretinal lesion with overlying vitritis without adjacent old scars (Figure *1*). 

The patient initially denied keeping kittens and eating raw or undercooked meat (lamb, ground beef, shellfish, game). The primary serologic survey was positive for cytomegalovirus (CMV), herpes simplex virus (HSV), varicella-zoster virus (VZV), and Toxoplasmosis IgG (and not IgM). The patient was immunocompetent (no neoplastic disease, HIV/AIDS, or undergoing immunomodulatory therapy). Chest radiography and angiotensin-converting enzyme (ACE) levels were normal. TB blood tests (IGRAs) and syphilis were negative.

More detailed history-taking revealed that the patient consumed grilled rats in Ghana. The ocular toxoplasmosis was assumed. Due to moderate vitreous involvement and segmental intravascular yellow-white Kyrieleis plaques in the central retinal artery, treatment with oral azithromycin 500 mg/day in five weeks started according to the local guidelines. After 48 hours, 1 mg/kg prednisolone was added to the treatment regime and tapered down in 10 days. The patient's best-corrected visual acuity fully recovered (BCVA: 20/20). No recurrence or new lesion was observed during the six-month follow-up period. 

## Discussion

In cases of uveitis, it's typically observed that intraocular pressure (IOP) tends to be low. However, in instances of acute hypertensive uveitis where IOP is elevated, healthcare providers should contemplate the possibility of an underlying infection, such as HSV, CMV, VZV, toxoplasmosis, or rubella. Furthermore, it is worth noting that other causes of hypertensive uveitis including Posner-Schlossman syndrome and Fuchs' heterochromic iridocyclitis are syndromes that may also be linked to these same infectious agents. Toxoplasma gondii, is one of the leading causes of chorioretinitis. Up to 20% of patients with ocular toxoplasmosis may experience an acute increase in intraocular pressure (IOP) [1].

Diagnosis of ocular toxoplasmosis is mainly clinical. A positive serologic test for anti-Toxoplasma gondii IgG or IgM antibodies serves as confirmation of exposure to the parasite. IgG antibodies typically emerge within the first two weeks of infection. When anti-Toxoplasma gondii IgG antibodies are present within the appropriate clinical context, they provide support for the diagnosis of toxoplasmic retinochoroiditis. Conversely, a negative antibody titer effectively rules out the diagnosis [1-3].

Although Kyrieleis arteritis has been described previously in earlier reports, diagnosis of atypical ocular toxoplasmosis can still be challenging. Moreover, in the current case, the choroidal lesion was located in the periphery and looks like a choroidal granuloma in the optical coherence tomography (OCT) B-scan (Figure 1B). It shows neither typical retinitis nor adjacent or nearby old scars. Segmental intravascular yellow-white Kyrieleis plaques were present. These plaques are most commonly reported in toxoplasmosis but can also be seen in other inflammatory, autoimmune, or infectious diseases (Table 1). Their pathogenesis is still highly debated. The plaques are not associated with periarterial or endoluminal injury, but the involvement of the arterial endothelium [2,3].

**Table 1:** Differential diagnosis of Kyrieleis arteritis or segmental retinal arteritis (SRA)

Diseases associated with Kyrieleis plaques or segmental retinal arteritis (SRA)
Behçet disease (BD)
Brolucizumab-associated retinal vasculitis
Cytomegalovirus (CMV)
Herpes simplex virus (HSV - 1 and 2)
Mycobacterium tuberculosis
Mediterranean spotted fever (Rickettsia conorii)
Sarcoidosis
Toxoplasma gondii
Treponema pallidum (Syphilis)
Varicella-zoster virus (VZV)

Kyrieleis plaques may need to be more reported. Kyrieleis arteritis should not confused with arterial emboli, frosted branch angiitis, or vascular sheathing. Their clinical and fluorescein angiographic features can differentiate these. They are not endothelial deposits or intraluminal which differentiates them from intraluminal arterial emboli. Kyrieleis plaques are highly reflective whitish-yellow glistening deposits along the outer walls of retinal arterioles. The high reflectivity of the vessel walls may confuse this with vascular sheathing. The appearance of frosted branch angiitis is different with more fluffy yellow-white changes along the vessels. Kyrieleis plaques affect only the retinal arteries in contrast to frosted branch angiitis which involves both the retinal arteries and veins. Fluorescein angiography (FA) shows no leakage or occlusions in the area of the plaques and no delay in the arterial filling. In contrast, frosted branch angiitis extends outside the vessel wall and extensively leaks fluorescein dye [3].

Although once most cases were believed to be the result of reactivation of congenital toxoplasmosis, it is now recognized that primary postnatally acquired infection accounts for many cases of this disease. The only definitive hosts for Toxoplasma gondii are members of the family Felidae including domestic cats. Intermediate hosts in nature include birds and rodents. Rats are eaten regularly in Cambodia, Laos, Myanmar, parts of the Philippines, Indonesia, Thailand, Ghana, China, and Vietnam [4]. The consumption of grilled rats as a source of infection helped the assumption of the diagnosis in the current case. This should be asked directly in history taking.

Treatment for ocular toxoplasmosis is not always necessary, as it tends to resolve on its own in the majority of cases. Systemic pyrimethamine, sulfadiazine, and corticosteroids are the classic triple therapy in toxoplasmosis. In Sweden, azithromycin 500 mg/day in five weeks is the routine regime used to treat ocular toxoplasmosis, if indicated [5]. The current case has shown a satisfactory response to this regime.

## Conclusions

Toxoplasmosis can present as multifocal arteritis with Kyrieleis plaques and peripheral choroidal elevation. Kyrieleis arteritis may be underrepresented. Diagnosis of atypical toxoplasmosis can be challenging. Eating rats can be a source of infection in parts of the world, which should be considered in history taking. Azithromycin 500 mg/day in five weeks is effective in control of atypical cases. 
